# Spatial Genetic Heterogeneity in Populations of a Newly Invasive Whitefly in China Revealed by a Nation-Wide Field Survey

**DOI:** 10.1371/journal.pone.0079997

**Published:** 2013-11-26

**Authors:** Dong Chu, Hui-Peng Pan, Xian-Chun Li, Dong Guo, Yun-Li Tao, Bai-Ming Liu, You-Jun Zhang

**Affiliations:** 1 Key Lab of Integrated Crop Pest Management of Shandong Province, College of Agronomy and Plant Protection, Qingdao Agricultural University, Qingdao, P. R. China; 2 Department of Plant Protection, Institute of Vegetables and Flowers, Chinese Academy of Agricultural Sciences, Beijing, P. R. China; 3 Department of Entomology and BIO5 Institute, University of Arizona, Tucson, Arizona, United States of America; College of Charleston, United States of America

## Abstract

**Background:**

Even though introductions of exotic species provide ready-made experiments of rapid evolution, few studies have examined the genetic structure of an exotic species shortly after its initial introduction and subsequent spread. To determine the genetic structure of its populations during the initial introduction, we investigated the invasive sweet potato whitefly (*Bemisia tabaci* Q, commonly known as *B. tabaci* biotype Q) in China, which was introduced in approximately 2003. A total of 619 *B. tabaci* Q individuals in 20 provinces throughout China were collected and analyzed using five microsatellite loci.

**Results:**

The introduced populations of *B. tabaci* Q in China represent eight genetic clusters with different geographic distributions. The populations in Yunnan Province, where *B. tabaci* Q was first detected, are genetically different from the other populations in China.

**Conclusion:**

The introduced populations of *B. tabaci* Q in China have high spatial genetic heterogeneity. Additional research is required to determine whether the heterogeneity results from multiple introductions, rapid evolution following one or few introductions, or some combination of multiple introductions and rapid evolution. The heterogeneity, however, is inconsistent with a single introduction at Yunnan Province, where *B. tabaci* Q was first detected, followed by spread.

## Introduction

The introduction of invasive species can cause substantial economic loss and ecological damage in the introduced regions [1], [2]. Invasions generally involve several ecological processes including initial introduction, establishment of a sustaining population, lag period, and range expansion. During these processes, the invasive species often evolve rapidly in response to novel abiotic and biotic conditions, and the characteristics of the rapid evolution of the introduced population is believed to be useful for understanding the potential for the invasion [3], [4]. The related studies on the genetic structure of the exotic species can provide novel insights into the evolution of the exotic species and its invasion pathways (e.g., [5], [6]).

Although many empirical studies on the genetic structure of invasive species (e.g., [7], [8]) have revealed that exotic species evolve, these studies have usually been done decades after the introduction. However, the genetic structure of exotic species during the early invasion period has rarely been explored [3], [9]. One of the most important reasons is that the processes of the initial introduction and subsequent spread are generally not well-documented. In most cases, the exotic species is not detected until its negative effects are severe and until it has spread throughout the introduced regions. Another important reason is that the invasions often go unrecognized for substantial periods because the introduced species are morphologically indistinguishable from native species or previously established species [10]. Improving our understanding of invasions will require additional analyses of the genetic structure of invasive species shortly after their initial introduction and subsequent spread.

The sweet potato whitefly, *Bemisia tabaci*, is an important agricultural pest that is distributed on all continents except Antarctica [11]. *Bemisia tabaci* has been regarded as a species complex that includes many morphologically indistinguishable biotypes that differ in host range, virus transmission, insecticide resistance, and other traits [11], [12]. Based on inter-biotype crosses and genetic differentiation using the mitochondrial cytochrome oxidase I (mtCOI) gene, recent studies suggest that many biotypes are cryptic species [12], [13]. Among them, *B. tabaci* Q (commonly known as biotype Q) has been introduced into many non-Mediterranean countries from its native Mediterranean regions over the past decade [13]. The genetic structure of indigenous *B. tabaci* populations has been analyzed on a large geographic scale (Asia-Pacific regions) [14] and on a small geographic scale (Greece) [15] using microsatellite loci. However, the genetic variation of the introduced populations of *B. tabaci* has been analyzed only rarely.

Here, we examined the genetic structure of *B. tabaci* Q populations in China. *Bemisia tabaci* Q, a cryptic species in the *B. tabaci* species complex [12], [13], was introduced into China in approximately 2003 [11]. Field surveys of whiteflies in 10 provinces of China in 2003 revealed that *B. tabaci* Q was present in Yunnan Province, Beijing, and Henan and that *B. tabaci* Q represented only a small percentage of the *B. tabaci* individuals detected [11]. In 2007, *B. tabaci* Q was detected in 19 of 22 whitefly populations in 15 surveyed provinces, and the percentage of *B. tabaci* Q individuals within 10 populations was higher than 50% [16]. During 2008–2009, *B. tabaci* Q was detected in 11 of 14 whitefly populations in eight surveyed provinces; of the 11 populations, nine were pure *B. tabaci* Q populations, and two were mixed populations of *B. tabaci* Q and B (commonly known as biotype B) [17]. During 2009–2010, *B. tabaci* Q was detected in 12 of 16 surveyed provinces [18]. In 2009, 44 of 61 populations collected from 19 surveyed provinces contained only *B. tabaci* Q [19]. These findings indicate that *B. tabaci* Q spread across China and became the dominant whitefly species in field ecosystems within approximately 4 years after its introduction. The well-documented invasion of *B. tabaci* Q in China or partial regions in China [20] offers an excellent opportunity to determine the genetic characteristics of an exotic species shortly after its introduction into a new habitat. We suspect that this rapid increase in the distribution and dominance of *B. tabaci* Q resulted from multiple introductions and subsequent spread rather than from a single introduction and subsequent spread.

The first objective of the present study was to determine the genetic structure of the introduced populations of *B. tabaci* Q across the entire country of China, and this was accomplished with microsatellite markers, which have been widely used in the analysis of invasive species [21]. The second and closely related objective was to use the genetic data to test the hypotheses that the rapid increase in the distribution and dominance of *B. tabaci* Q in China resulted from a single introduction (at Yunnan Province, where *B. tabaci* Q was first detected) and subsequent spread vs. multiple introductions and subsequent spread.

## Materials and Methods

### Ethics Statement

The research complies with all laws of the country (China) in which it was performed and was approved by the ‘Department of Scientific Management of Chinese Academy of Agricultural Sciences, China’ (permit number: 20090112).

### Field sampling

**Figure 1:**
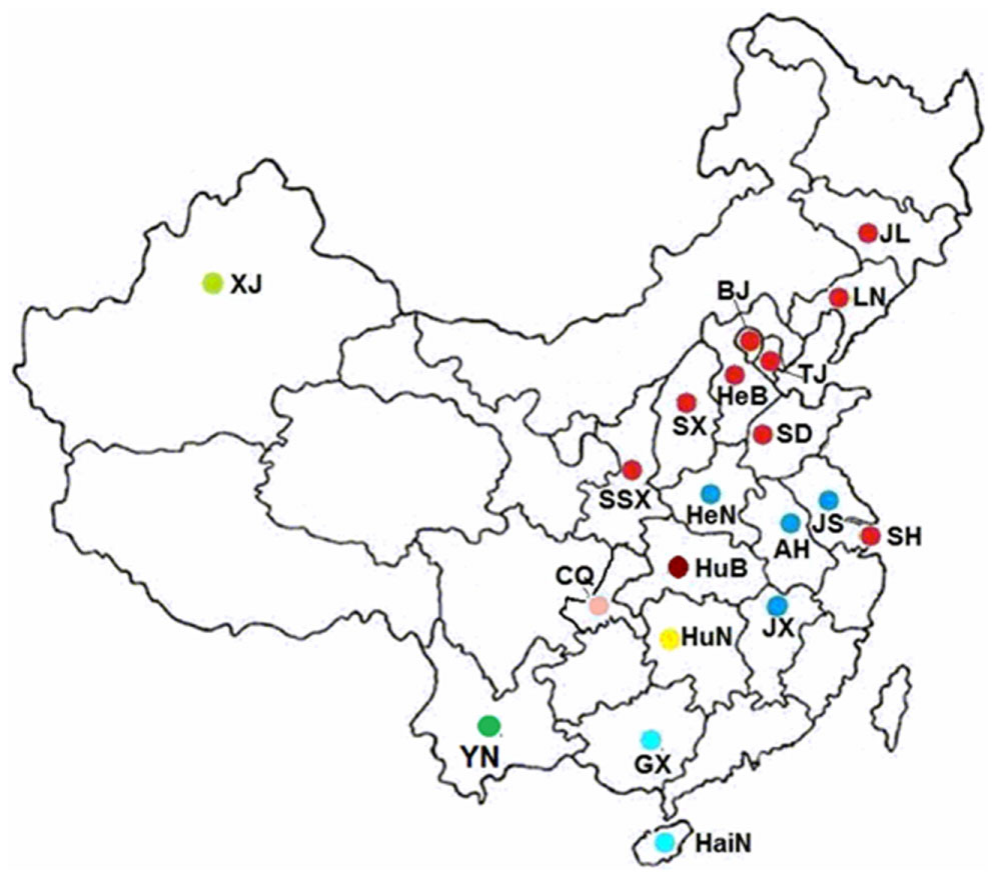
Location of Chinese populations of *B. tabaci* Q in this study. Eight clusters identified by BAPS (see text and Fig. 2): cluster I consisted of the populations indicated by green font (JL, LN, BJ, TJ, HeB, SX, SSX, SD, and SH); cluster II consisted of the populations indicated by red font (HeN, JS, AH, and JX); cluster III consisted of the populations indicated by yellow font (GX and HaiN); and cluster IV, V, VI, VII, and VIII each consisted of one population (CQ, XJ, HuB, HuN, and YN, respectively).

Adult *B. tabaci* were collected from a variety of field crops (e.g., cotton, tomato, cucumber, and eggplant) and weeds (e.g., Japanese hop) in 25 provinces of China in 2011–2012 (Fig. 1). We found *B. tabaci* Q in 22 of 25 surveyed provinces (unpublished data), and the specimens of *B. tabaci* Q from 20 provinces were used in the present study. At least 100 living specimens were collected from the major host plants at each site (host plants are listed in Table 1). The specimens were fixed in 95% ethanol and stored at −20°C until DNA was extracted.

**Table 1:** Site characteristics and genetic diversity indices of *B. tabaci* Q populations.

Locality	Code	Date	Host	*N*	*Na*	*Ne*	*Ho*	*He*	*Nei*	*Fis*	*Pwil*
Haidian, Beijing	BJ	2011.8	Eggplant, tomato, cotton	42	4.80	2.0739	0.2762	0.4420	0.4367	**0.3780**	**0.0313**
Shijiazhuang, Hebei	HeB	2011.8	Eggplant, tomato, cotton	35	3.80	2.3014	0.2649	0.4841	0.4771	**0.4576**	0.8906
Changchun, Jilin	JL	2011.8	Eggplant, tomato	45	4.60	2.2124	0.3627	0.4830	0.4776	**0.2502**	0.4063
Yuncheng, Shanxi	SX	2011.8	Eggplant, cotton	23	4.40	2.6189	0.2917	0.5034	0.4923	**0.4247**	0.4063
Yangling, Shanxi	SSX	2011.8	Eggplant, tomato, cotton	36	5.60	2.0976	0.4285	0.4306	0.4244	**0.1766**	0.4063
Minhang, Shanghai	SH	2011.8	Eggplant, tomato	30	4.80	2.2474	0.2692	0.4641	0.4563	**0.4248**	**0.0469**
Zhongan, Liaoning	LN	2011.8	Eggplant, tomato, cotton	18	4.60	2.5085	0.3778	0.5613	0.5457	**0.3333**	0.3125
Jinan, Shandong	SD	2011.8	Eggplant, tomato, Japanese hop	43	5.00	2.2808	0.3628	0.4909	0.4852	**0.2633**	0.1094
Wuhe, Tianjin	TJ	2011.8	Tomato	11	3.60	2.1810	0.2545	0.4597	0.4388	**0.4584**	0.1094
Sanshigang, Anhui	AH	2011.8	Eggplant, tomato, cotton	28	5.20	2.3981	0.2672	0.4830	0.4743	**0.4498**	**0.0469**
Luoyang, Henan	HeN	2011.8	Tomato, cotton	11	3.80	2.2943	0.2727	0.4987	0.4760	**0.4652**	0.1563
Nanjing, Jiangsu	JS	2011.8	Eggplant, cotton, tomato	45	5.20	2.5804	0.4698	0.5665	0.5601	**0.1724**	0.6875
Nanchang, Jiangxi	JX	2011.8	Eggplant, tomato	30	4.80	2.2853	0.3069	0.4746	0.4666	**0.3530**	0.1094
Nanning, Guangxi	GX	2011.8	Cucumber, tomato	20	4.20	2.5676	0.3600	0.4946	0.4822	**0.2773**	0.4063
Beipei, Chongqing	CQ	2011.8	Eggplant, tomato	29	4.20	2.8972	0.2916	0.5642	0.5544	**0.4886**	0.8906
Haikou, Hainan	HaiN	2011.8	Eggplant, tomato	21	4.40	2.4917	0.2762	0.5189	0.5065	**0.4730**	0.3125
Changsha, Hunan	HuN	2011.8	Eggplant, tomato, cotton	42	5.80	2.7371	0.2158	0.5247	0.5183	**0.5945**	0.1094
Wuhan, Hubei	HuB	2011.8	Eggplant, tomato, cotton	45	5.20	3.0546	0.4432	0.6004	0.5937	**0.2650**	0.8906
Tulufan, Xinjiang	XJ	2011.8	Eggplant, tomato, cotton	39	4.20	2.5042	0.4333	0.5410	0.5341	**0.1896**	0.8906
Kunming, Yunnan	YN	2012.8	Eggplant, pepper	26	3.20	1.9330	0.3846	0.3977	0.3901	**0.0336**	0.8438
Mean±SD				31.0±11.2	4.57±0.67	2.4133±0.2806	0.3305±0.0741	0.4992±0.0504	0.4895±0.0504	0.3464±0.1396	
Total				619	10.60	2.8389	0.3405	0.5672	0.5668	0.3431	

For each sample, the following are indicated: sampling site, population code, date of collection, host plant, sample size (*N*), average number of alleles per locus (*Na*), the effective number of alleles (*Ne*), the observed heterozygosity (*Ho*), the expected heterozygosity (*He*), and Nei's expected heterozygosity (*Nei*), estimator of the fixation index (*Fis*), and the Wilcoxon test *P* value for heterozygosity deficit compared to expectations at mutation-drift equilibrium (*Pwil*). Significant values for *Fis* and for heterozygosity deficiency are in bold.

### DNA extraction and microsatellite genotyping

Genomic DNA was individually extracted from each of the collected adult whiteflies using the DNAzol kit (Molecular Research Center, Inc., Cincinnati, OH) and stored at −20°C. The mtCOI gene sequence was used to determine the species of *B. tabaci*. All individual DNA samples were amplified using the mtCOI primers C1-J-2195 (5′-TTGATTTTTTGGTCATCCAGAAGT-3′) and L2-N-3014 (5′-TCCAATGCACTAATC TGCCATATTA-3′) and then sequenced [22]. These sequences were aligned with Clustal W [23] and were then checked for indels and numts. The unknown sequences were compared against the consensus sequences for each of the 24 putative species identified by Dinsdale *et al*. [12]. These unknown sequences were regarded as *B. tabaci* Q if their divergence from the consensus sequence of *B. tabaci* Q (the sequence is labeled as MED by Dinsdale *et al*. [12]) was <3.5%. The species of 15 whitefly individuals each host population was determined. Finally, a total of 619 individuals were determined to be *B. tabaci* Q in this study.

PCR primers were used to amplify the microsatellite DNA loci (BEM06, BEM11, BEM25, BEM31, and BEM37) using the DNA of *B. tabaci* Q individuals as template [24]. PCR reactions were performed as described in De Barro *et al*. [24], and products were run on an ABI 3730xl DNA analyzer. Allele size was determined by comparing the mobility of the PCR products to that of the GeneScanTM 400HD size standard (Applied Biosystems).

### Analyses of genetic diversity

For each of the 20 populations of *B. tabaci* Q, the average number of alleles per locus (*Na*), the effective number of alleles (*Ne*), the observed heterozygosity (*Ho*), the expected heterozygosity (*He*), and Nei's expected heterozygosity (*Nei*) were calculated using POPGENE v.1.31 [25].

### Analyses of genetic structure within populations

The Wahlund effect within each population was quantified by calculating the Weir and Cockerham's *Fis* [26], a multilocus estimator of the fixation index, with GENEPOP v.3.4 software [27]. Conformity to Hardy–Weinberg equilibrium was assessed with exact tests in GENEPOP v.3.4 with Markov chain parameters of 10,000 dememorization steps, followed by 1,000 batches of 10,000 iterations per batch. We tested for deviation from mutation-drift equilibrium in the populations using the approach in BOTTLENECK software [28]. The heterozygosity deficit may provide evidence for population expansion while the heterozygosity excess may provide evidence for a genetic bottleneck. Using a Wilcoxon test, we evaluated the heterozygosity deficit under the two-phase mutation model (TPM) recommended for microsatellite data [29] using BOTTLENECK software. The possibility of bottleneck events within the 20 populations was examined using BOTTLENECK software under three mutation models [Two Phase Mutation Model (TPM), Infinite Allele Model (IAM), and Stepwise Mutation Model (SMM)] [28], [29]. The TPM model was used with the default settings of 30% variation from the IAM model and 70% from the SMM model.

### Analyses of genetic structure among populations

We used a traditional population differentiation approach based on *Fst* values. Weir and Cockerham's estimator of the fixation index *Fst* [26] was calculated with GENEPOP v.3.4 [27].

The program BAPS v.4.14 [30], [31] was used to detect clusters of the *B. tabaci* Q populations in China and to estimate individual coefficients of ancestry with regard to the detected clusters. When estimating individual ancestry coefficients via admixture analyses, we used the recommended values: the number of iterations used to estimate the admixture coefficients for the individuals was 100; the number of reference individuals from each population was 200; and the number of iterations used to estimate the admixture coefficients for the reference individuals was 20.

To estimate the variance among clusters, among populations within clusters, and within clusters, a hierarchical analysis of molecular variance (AMOVA) was performed with ARLEQUIN v.3.5 [32]. Microsatellite data were partitioned to enable a comparison of variation among populations and among individuals within populations. AMOVA computations were performed with 10,000 permutations to test for significance.

## Results

### Genetic diversity

Values of genetic diversity indices of the Chinese populations are given in Table 1. The average number of alleles per locus (*Na*) ranged from 3.20 to 5.80, and the effective number of alleles (*Ne*) ranged from 1.9330 to 3.0546. The expected heterozygosity (*He*) ranged from 0.3977 to 0.6004 while the observed heterozygosity (*Ho*) ranged from 0.2158 to 0.4698. The *He* in each population was higher than the value of *Ho*. Nei's expected heterozygosity (*Nei*) ranged from 0.3901 to 0.5937. The levels of genetic diversity indices were similar but not always consistent. For instance, population YN had the lowest level of genetic diversity according to *Na* (3.20), *Ne* (1.9330), *He* (0.3977), and *Nei* (0.3901) but not according to *Ho* (0.3846).

### Analyses of genetic structure within populations

The estimator of the fixation index, *Fis*, demonstrated the presence of sub-structure within all populations (Table 1; Table S1). In testing for deviation from mutation-drift equilibrium in BOTTLENECK, we detected a significant heterozygosity deficit (Wilcoxon test *P*<0.05) in only three populations (BJ, SH, and AH), which account for 15% of the Chinese populations. The significant heterozygosity deficit for the three populations, however, may result not from demographic expansion but rather from within-population substructure [21], [28]. In support of this interpretation, significant departures from Hardy–Weinberg equilibrium (*Fis*) in these populations (Table 1; Table S1) suggest that the significant deviation from mutation-drift equilibrium may be due to sub-structure (the Wahlund effect) within these localities.

In testing for deviation from mutation-drift equilibrium in BOTTLENECK, we did not detect a significant heterozygosity excess in any population under the TPM model or the SMM model. Under the IAM model, a significant heterozygosity excess (Wilcoxon test *P*<0.05) was detected in only four populations (JS, CQ, HuB, and AH) (Table 2), indicating that these four populations may have experienced a genetic bottleneck (Table 1).

**Table 2:** Within-population tests for heterozygosity excess *P*-values according to three models (IAM, TPM, and SMM).

Locality	Population code	Heterozygosity excess *P*-values		
		IAM	TPM	SMM
Haidian, Beijing	BJ	0.59373	0.98438	1.00000
Shijiazhuang, Hebei	HeB	0.10938	0.31250	0.92188
Changchun, Jilin	JL	0.40625	0.68750	0.98438
Yuncheng, Shanxi	SX	0.31250	0.68750	0.95313
Yangling, Shanxi	SSX	0.50000	0.68750	0.95313
Minhang, Shanghai	SH	0.89063	0.96875	1.00000
Zhongan, Liaoning	LN	0.40625	0.89063	0.96875
Jinan, Shandong	SD	0.59375	0.92188	0.98438
Wuhe, Tianjin	TJ	0.59375	0.92188	0.96875
Sanshigang, Anhui	AH	0.89063	0.96875	1.00000
Luoyang, Henan	HeN	0.09375	0.90625	0.96875
Nanjing, Jiangsu	JS	**0.04688**	0.40625	0.95313
Nanchang, Jiangxi	JX	0.68750	0.92188	0.96875
Nanning, Guangxi	GX	0.59375	0.68750	0.68750
Beipei, Chongqing	CQ	**0.04688**	0.31250	0.40625
Haikou, Hainan	HaiN	0.31250	0.89063	0.96875
Changsha, Hunan	HuN	0.50000	0.92188	0.96875
Wuhan, Hubei	HuB	**0.03125**	0.31250	0.59375
Tulufan, Xinjiang	XJ	**0.04688**	0.31250	0.59375
Kunming, Yunnan	YN	0.09375	0.43750	0.90625

Bold indicates significance at *P*<0.05.

### Analyses of genetic structure among populations

**Figure 2:**
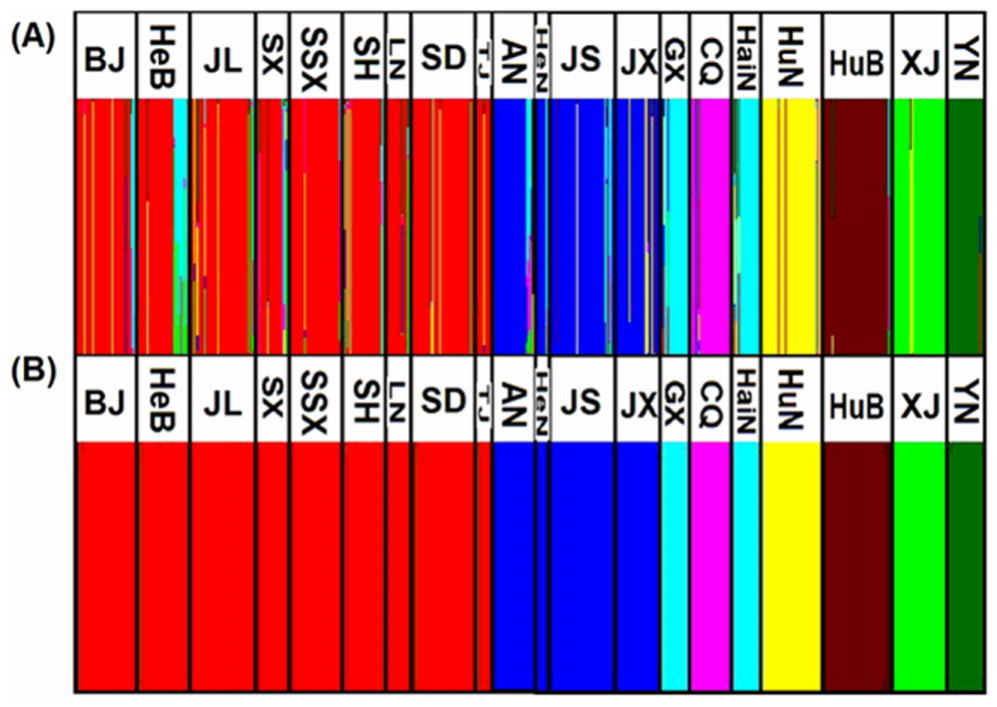
Bayesian individual clustering and Bayesian population clustering for the microsatellite data set of *B. tabaci* Q in China. In both A and B, individuals are grouped by sampling location. Abbreviations shown at the top of panels A and B are code names of sampling locations as indicated in Figure 1. (A) Each individual is represented by a vertical bar partitioned into colored segments using BAPS. (B) Delineation of eight genetic clusters using BAPS. Each color indicates sampling localities belonging to each cluster.

When considering each population pair, 153 of 190 (81%) *Fst* values were associated with a significant exact test (Table 3). The values of *Fst* in comparisons of the population from Yunnan (YN) and other populations ranged from 0.3622 to 0.4937, which were much higher than other *Fst* values (<0.2366). The main results from *Fst* computations were confirmed by Bayesian analyses. Analyses using BAPS identified eight genetic clusters overall (Fig. 2): one cluster consisted of nine populations (JL, LN, BJ, TJ, HeB, SX, SSX, SD, and SH); one cluster consisted of four populations (HeN, JS, AH, and JX); one cluster consisted of two populations (GX and HaiN); and each of the other five clusters consisted of one population (XJ, HuB, HuN, CQ, and YN).

**Table 3:** Population pairwise *Fst*.

Population code	BJ	HeB	JL	SX	SSX	SH	LN	SD	TJ	AH	HeN	JS	JX	GX	CQ	HaiN	HuN	HuB	XJ
HeB	**0.0295**																		
JL	**−0.0023**	**0.0174**																	
SX	**0.0579**	**0.0248**	**0.0331**																
SSX	**0.0620**	**0.0547**	**0.0422**	**0.0334**															
SH	0.0102	**0.0164**	0.0087	0.0119	0.0119														
LN	**0.0291**	**0.0030**	−0.0005	0.0182	**0.0692**	0.0305													
SD	**0.0250**	**0.0234**	0.0064	0.0114	0.0059	0.0011	0.0173												
TJ	−0.0098	**0.0107**	−0.0137	0.0109	−0.0005	−0.0183	0.0154	−0.0195											
AH	**0.0219**	**0.0366**	**0.0225**	**0.0510**	**0.0858**	**0.0202**	**0.0414**	**0.0516**	0.0132										
HeN	0.0058	0.0057	0.0063	0.0139	**0.0457**	−0.0213	0.0114	0.0155	−0.0101	−0.0072									
JS	**0.0427**	**0.0439**	**0.0333**	**0.0509**	**0.0589**	**0.0244**	**0.0386**	**0.0328**	0.0166	**0.0304**	0.0119								
JX	**0.0122**	**0.0343**	**0.0211**	**0.0446**	**0.0590**	0.0022	**0.0484**	0.0223	−0.0038	**0.0239**	−0.0172	**0.0178**							
GX	**0.1331**	**0.0772**	**0.1288**	**0.0909**	**0.1721**	**0.1011**	**0.0992**	**0.1250**	**0.1295**	**0.0999**	**0.0793**	**0.0875**	**0.0960**						
CQ	**0.0900**	**0.0422**	**0.0809**	**0.0537**	**0.1222**	**0.0735**	**0.0417**	**0.0831**	**0.0797**	**0.0809**	**0.0560**	**0.0640**	**0.0898**	**0.0322**					
HaiN	**0.1812**	**0.1182**	**0.1723**	**0.1605**	**0.2366**	**0.1680**	**0.1224**	**0.1735**	**0.1787**	**0.1706**	**0.1422**	**0.1194**	**0.1493**	0.0166	**0.0493**				
HuN	**0.1259**	**0.1319**	**0.1235**	**0.1392**	**0.2154**	**0.1531**	**0.0984**	**0.1534**	**0.1310**	**0.1557**	**0.1021**	**0.1328**	**0.1049**	**0.1841**	**0.1499**	**0.1956**			
HuB	**0.0773**	**0.0748**	**0.0703**	**0.0740**	**0.1331**	**0.0832**	**0.0546**	**0.0793**	**0.0657**	**0.0767**	**0.0468**	**0.0532**	**0.0403**	**0.1107**	**0.0965**	**0.1331**	**0.0296**		
XJ	**0.0869**	**0.0177**	**0.0565**	**0.0538**	**0.1039**	**0.0718**	0.0121	**0.0609**	**0.0598**	**0.0724**	**0.0530**	**0.0511**	**0.0859**	**0.0912**	**0.0438**	**0.1110**	**0.1480**	**0.0938**	
YN	**0.4717**	**0.4338**	**0.4419**	**0.4379**	**0.4937**	**0.4641**	**0.3954**	**0.4414**	**0.4837**	**0.4702**	**0.4648**	**0.3969**	**0.4612**	**0.4211**	**0.3622**	**0.3722**	**0.4436**	**0.3859**	**0.3998**

Significant values for pairwise *Fst* are in bold.

Significant genetic structure of *B. tabaci* Q was observed at three hierarchical levels (among clusters, among populations within clusters, and within clusters) (Table 4). Most of the variation was within clusters (86.03%), and both the variation among populations within clusters (1.99%, *F_SC_* = 0.02266, *P*<0.0001) and the variation among clusters (11.97%, *F_CT_* = 0.11973, *P*<0.0001) were small but significant.

**Table 4:** Analysis of molecular variance (AMOVA) for population structures of *Bemisia tabaci* Q.

Source of variation	*d.f.*	Sum of squares	Variance components	Percentage of variation	Fixation indices
Among clusters	7	185.492	0.17761 Va	11.97	*F_CT_* = 0.11973 (*P*<0.0001)
Among populations within clusters	12	36.019	0.02959 Vb	1.99	*F_SC_* = 0.02266 (*P*<0.0001)
Within clusters	1224	1562.076	1.27621 Vc	86.03	*F_ST_* = 0.13968 (*P*<0.0001)
Total	1243	1783.588	1.48340		

## Discussion

To the best of our knowledge, this is the first study to report the genetic structure of *B. tabaci* Q in China at the national level. Our results show that all Chinese populations have sub-structure even though *B. tabaci* Q was only recently introduced into the country. Most microsatellites fit a two-phase model of mutation (TPM) better than a strict stepwise mutation model (SMM) or infinite alleles model (IAM) [29]. Based on the TPM model, only a few Chinese populations (BJ, SH, and AH) significantly depart from Hardy–Weinberg equilibrium. As noted in the Results, the significant departures from Hardy–Weinberg equilibrium observed in these three populations may be due to sub-structure (the Wahlund effect) within these localities rather than to demographic expansion.

The *Fst* data and analyses using BAPS show that the introduced populations are genetically different. There are many possible explanations for the genetic heterogeneity of *B. tabaci* Q populations in China. One possible explanation is that there have been multiple introductions of exotic *B. tabaci* Q in China, with diversity in the genotypes among introductions. The second possibility is rapid evolution following only one or a few introductions. A third possibility would include both multiple introductions and rapid evolution. With regard to rapid evolution, both genetic drift and natural selection (from biotic interactions and abiotic factors in the new environment) may result in genetic heterogeneity of the introduced populations [33]–[36]. For example, the application of insecticides can enhance genetic differentiation by resulting in bottleneck effects [34]–[36].

Our study suggests (but does not prove) that bottleneck effects did not play an important role during the genetic differentiation of *B. tabaci* Q in China because only four of the 20 populations exhibited significant heterozygosity excess (a signature of a bottleneck) under the TPM model, and none exhibited heterozygosity excess under either the IAM or SMM model. The effects of a bottleneck on heterozygosity are transient and observable only for a few generations [37]. In most of China, *B. tabaci* generally has 11–15 generations each year (http://www.ipm.ioz.ac.cn/them_gefeng/fenshi/yuce.asp). Thus, approximately 88–120 generations have occurred since the introduction of Q in about 2003 [11], which is sufficient to obscure the effects of a bottleneck. The genetic effects of a bottleneck during the period might also be obscured by the high gene flow among the introduced populations [38]. The changes in genetic diversity also support the inference that the effects of a bottleneck on introduced *B. tabaci* Q populations have been mitigated. The nuclear genetic diversity of introduced populations of *B. tabaci* Q in China (*He* range: 0.3977–0.6004) in this study is similar to that of *B. tabaci* Q in native regions (*He* range: 0.3594–0.6124) as reported by Chu *et al*. [39].

We also cannot exclude the effects of human activities on the genetic heterogeneity of the introduced populations. The frequent transportation of ornamental plants or crop seedlings within a province or between neighboring provinces enhances gene flow between populations. Bayesian analyses indicated that the gene flow among the neighboring provinces is variable. These data suggest that the initial populations of *B. tabaci* Q, especially those in northern China, have spread to nearby provinces by natural expansion or human activity. For example, the populations in cluster I (except SH) are mainly composed of the populations in northern China including populations in nine neighboring provinces (Jilin, Liaoning, Beijing, Tianjin, Shandong, Hebei, Shanxi, and Shaanxi) (Fig. 2). Cluster II is mainly composed of the populations in four neighboring provinces including Henan, Anhui, Jiangsu, and Jiangxi provinces (Fig. 2). Cluster III is mainly composed of the populations in Hainan and Guangxi provinces, which are not geographically connected but are very close (Fig. 2). Although the other five clusters are composed of single populations, each from only one province, the five clusters do not indicate that gene flow between these provinces is limited. The five single-population clusters could be explained by rapid genetic turnover of the *B. tabaci* Q populations in these regions, because a recent study documented significant temporal change in local genetic composition accompanied by heterozygosity deficits and inbreeding in *B. tabaci* B [33].

*B. tabaci* Q was first detected in the USA [40], but long-term monitoring of *B. tabaci* Q in field and greenhouse systems showed that it cannot establish self-sustaining populations in the field in the USA [41], [42]. The different invasion patterns in the USA and China suggest that USA populations of *B. tabaci* Q have limited invasiveness and that the genetic composition of *B. tabaci* Q populations differs in the USA and China. It seems, therefore, that the *B. tabaci* Q populations in China were not derived from those in the USA.

Our data on genetic diversity and differentiation also suggest that *B. tabaci* Q populations in other provinces of China are not derived from populations in Yunnan Province, where the pest was first detected, even though infested ornamental plants and flowers are likely to be transported from Yunnan Province to the other provinces (http://www.accci.com.au/keycity/yunnan.htm). The introduced populations in Yunnan Province may not have acted as bridgehead population [38], [43] for two reasons. First, the genetic diversity in the Yunnan population (YN) is the lowest among Chinese populations. Second, the high *Fst* value indicates that the Yunnan population is highly differentiated from the other populations.

## Conclusions

Although introductions of exotic species have provided excellent opportunities to investigate rapid evolution, few studies have examined the genetic structure of an exotic species shortly after its initial introduction and subsequent spread. In this study, we found eight genetic clusters among the introduced populations of *B. tabaci* Q in China, which demonstrates that the introduced populations of *B. tabaci* Q in China have high spatial genetic heterogeneity. The heterogeneity may have resulted from a combination of multiple introductions of diverse populations and rapid evolution following introduction. Determining the relative contributions of these two sources of heterogeneity will require additional research.

## Supporting Information

Table S1 **Summary statistics for five microsatellite loci screened for** ***Bemisia tabaci*** **Q.** Abbreviations are as follows: sample size (*N*), number of alleles observed per locus (*Na*), observed heterozygosities (*Ho*) and expected heterozygosities (*He*), Wright's fixation index (*Fis*). *Fis* values in bold indicate significant departures from Hardy–Weinberg proportions.
(DOC)
