# Supplementary material for: Spatial Genetic Heterogeneity in Populations of a Newly Invasive Whitefly in China Revealed by a Nation-Wide Field Survey
Source: PLoS One. 2013 Nov 26;8(11):e79997. doi: 10.1371/journal.pone.0079997 (PMC3841195; doi:10.1371/journal.pone.0079997)
Supplement: Table S1 — Summary statistics for five microsatellite loci screened for Bemisia tabaci Q. Abbreviations are as follows: sample size (N), number of alleles observed per locus (Na), observed heterozygosities (Ho) and expected heterozygosities (He), Wright's fixation index (Fis). Fis values in bold indicate significant departures from Hardy–Weinberg proportions. (DOC) [file pone.0079997.s001.doc]

**Table S1** Summary statistics for five microsatellite loci screened for *Bemisia tabaci* Q. Abbreviations are as follows: sample size (*N*), number of alleles observed per locus (*Na*), observed (*Ho*) and expected (*He*), heterozygosities, Wright’s fixation index (*Fis*). *Fis* values in bold indicate significant departures from Hardy–Weinberg proportions.

|  |  | BEM6 | BEM11 | BEM25 | BEM31 | BEM37 | All loci |
| --- | --- | --- | --- | --- | --- | --- | --- |
| BJ |  |  |  |  |  |  |  |
|  | *N* | 84 | 84 | 84 | 84 | 84 | 84 |
|  | *Na* | 4.0000 | 4.0000 | 9.0000 | 4.0000 | 3.0000 | 4.8 (24) |
|  | *Ho* | 0.0476 | 0.4048 | 0.5000 | 0.4048 | 0.0238 | 0.2762 |
|  | *He* | 0.4759 | 0.3904 | 0.7338 | 0.5396 | 0.0703 | 0.4420 |
|  | *Fis* | **0.9010** | -0.0372 | **0.3212** | **0.2521** | **0.6639** | **0.3780** |
| HeB |  |  |  |  |  |  |  |
|  | *N* | 70 | 68 | 70 | 70 | 68 | 69 |
|  | *Na* | 4.0000 | 3.0000 | 6.0000 | 4.0000 | 2.0000 | 3.8 (19) |
|  | *Ho* | 0.0286 | 0.3235 | 0.4571 | 0.4857 | 0.0294 | 0.2649 |
|  | *He* | 0.5536 | 0.4298 | 0.7172 | 0.6903 | 0.0294 | 0.4841 |
|  | *Fis* | **0.9491** | 0.2500 | **0.3660** | **0.2994** | -0.0000 | **0.4576** |
| JL |  |  |  |  |  |  |  |
|  | *N* | 88 | 88 | 90 | 90 | 90 | 89 |
|  | *Na* | 5.0000 | 3.0000 | 8.0000 | 4.0000 | 3.0000 | 4.6 (23) |
|  | *Ho* | 0.0455 | 0.5682 | 0.5556 | 0.4889 | 0.1556 | 0.3627 |
|  | *He* | 0.4702 | 0.4830 | 0.7471 | 0.5680 | 0.1466 | 0.4830 |
|  | *Fis* | **0.9043** | -0.1787 | **0.2585** | 0.1407 | -0.0621 | **0.2502** |
| SX |  |  |  |  |  |  |  |
|  | *N* | 44 | 44 | 46 | 46 | 46 | 45 |
|  | *Na* | 5.0000 | 4.0000 | 8.0000 | 3.0000 | 2.0000 | 4.4 (22) |
|  | *Ho* | 0.0000 | 0.5455 | 0.5217 | 0.3478 | 0.0435 | 0.2917 |
|  | *He* | 0.5624 | 0.5180 | 0.8406 | 0.5527 | 0.0435 | 0.5034 |
|  | *Fis* | **1.0000** | -0.0544 | **0.3846** | 0.3759 | -0.0000 | **0.4247** |
| SSX |  |  |  |  |  |  |  |
|  | *N* | 72 | 72 | 70 | 70 | 68 | 70 |
|  | *Na* | 2.0000 | 2.0000 | 9.0000 | 3.0000 | 12.0000 | 5.6 (28) |
|  | *Ho* | 0.0000 | 0.3611 | 0.7429 | 0.6857 | 0.3529 | 0.4285 |
|  | *He* | 0.1064 | 0.3940 | 0.7694 | 0.5130 | 0.3701 | 0.4306 |
|  | *Fis* | **0.7929** | -0.0024 | **0.2668** | **-0.0047** | **0.4864** | **0.1766** |
| SH |  |  |  |  |  |  |  |
|  | *N* | 60 | 60 | 60 | 58 | 60 | 60 |
|  | *Na* | 4.0000 | 5.0000 | 7.0000 | 5.0000 | 3.0000 | 4.8 (24) |
|  | *Ho* | 0.0333 | 0.3333 | 0.5333 | 0.3793 | 0.0667 | 0.2692 |
|  | *He* | 0.3734 | 0.4842 | 0.7797 | 0.5856 | 0.0977 | 0.4641 |
|  | *Fis* | **0.9121** | **0.3152** | **0.3196** | **0.3563** | 0.3216 | **0.4248** |
| LN |  |  |  |  |  |  |  |
|  | *N* | 36 | 36 | 36 | 36 | 36 | 36 |
|  | *Na* | 6.0000 | 4.0000 | 6.0000 | 4.0000 | 3.0000 | 4.6 (23) |
|  | *Ho* | 0.2778 | 0.3889 | 0.4444 | 0.6667 | 0.1111 | 0.3778 |
|  | *He* | 0.6556 | 0.5429 | 0.7508 | 0.6492 | 0.2079 | 0.5613 |
|  | *Fis* | **0.5833** | 0.2896 | **0.4151** | -0.0277 | 0.4729 | **0.3333** |
| SD |  |  |  |  |  |  |  |
|  | *N* | 86 | 86 | 86 | 86 | 86 | 86 |
|  | *Na* | 5.0000 | 5.0000 | 8.0000 | 4.0000 | 3.0000 | 5.0 (25) |
|  | *Ho* | 0.0233 | 0.4884 | 0.6977 | 0.5116 | 0.0930 | 0.3628 |
|  | *He* | 0.3948 | 0.5387 | 0.7513 | 0.6170 | 0.1529 | 0.4909 |
|  | *Fis* | **0.9417** | **0.0945** | 0.0722 | **0.1724** | **0.3946** | **0.2633** |
| TJ |  |  |  |  |  |  |  |
|  | *N* | 22 | 22 | 22 | 22 | 22 | 22 |
|  | *Na* | 3.0000 | 3.0000 | 7.0000 | 3.0000 | 2.0000 | 3.6 (18) |
|  | *Ho* | 0.0909 | 0.2727 | 0.4545 | 0.3636 | 0.0909 | 0.2545 |
|  | *He* | 0.3247 | 0.4978 | 0.7965 | 0.5887 | 0.0909 | 0.4597 |
|  | *Fis* | **0.7297** | **0.4643** | **0.4413** | 0.3939 | -0.0000 | **0.4584** |
| AH |  |  |  |  |  |  |  |
|  | *N* | 54 | 54 | 56 | 56 | 56 | 55 |
|  | *Na* | 5.0000 | 6.0000 | 9.0000 | 4.0000 | 2.0000 | 5.2 (26) |
|  | *Ho* | 0.0370 | 0.3704 | 0.5357 | 0.2500 | 0.1429 | 0.2672 |
|  | *He* | 0.4647 | 0.4640 | 0.8169 | 0.5344 | 0.1351 | 0.4830 |
|  | *Fis* | **0.9217** | **0.2049** | **0.3484** | **0.5368** | -0.0588 | **0.4498** |
| HeN |  |  |  |  |  |  |  |
|  | *N* | 22 | 22 | 22 | 22 | 22 | 22 |
|  | *Na* | 4.0000 | 3.0000 | 7.0000 | 4.0000 | 1.0000 | 3.8 (19) |
|  | *Ho* | 0.0909 | 0.2727 | 0.4545 | 0.5455 | 0.0000 | 0.2727 |
|  | *He* | 0.5671 | 0.5065 | 0.7619 | 0.6580 | 0.0000 | 0.4987 |
|  | *Fis* | **0.8462** | 0.4737 | **0.4152** | 0.1781 | - | **0.4652** |
| JS |  |  |  |  |  |  |  |
|  | *N* | 90 | 86 | 88 | 90 | 88 | 88 |
|  | *Na* | 6.0000 | 4.0000 | 10.0000 | 4.0000 | 2.0000 | 5.2 (26) |
|  | *Ho* | 0.1333 | 0.3023 | 0.7727 | 0.8222 | 0.3182 | 0.4698 |
|  | *He* | 0.5745 | 0.4364 | 0.7861 | 0.6345 | 0.4013 | 0.5665 |
|  | *Fis* | **0.7699** | **0.3097** | **0.0171** | -0.3003 | 0.2089 | **0.1724** |
| JX |  |  |  |  |  |  |  |
|  | *N* | 58 | 60 | 60 | 60 | 60 | 60 |
|  | *Na* | 5.0000 | 5.0000 | 9.0000 | 3.0000 | 2.0000 | 4.8 (24) |
|  | *Ho* | 0.0345 | 0.2333 | 0.5667 | 0.5667 | 0.1333 | 0.3069 |
|  | *He* | 0.5233 | 0.3294 | 0.7740 | 0.6198 | 0.1266 | 0.4746 |
|  | *Fis* | **0.9352** | **0.2951** | 0.2712 | 0.0870 | -0.0545 | **0.3530** |
| GX |  |  |  |  |  |  |  |
|  | *N* | 40 | 40 | 40 | 40 | 40 | 40 |
|  | *Na* | 4.0000 | 4.0000 | 7.0000 | 3.0000 | 3.0000 | 4.2 (21) |
|  | *Ho* | 0.0500 | 0.1000 | 0.6500 | 0.8500 | 0.1500 | 0.3600 |
|  | *He* | 0.6603 | 0.1910 | 0.8115 | 0.6654 | 0.1449 | 0.4946 |
|  | *Fis* | **0.9261** | **0.4830** | 0.2032 | -0.2869 | -0.0364 | **0.2773** |
| CQ |  |  |  |  |  |  |  |
|  | *N* | 58 | 56 | 58 | 58 | 58 | 58 |
|  | *Na* | 6.0000 | 2.0000 | 6.0000 | 4.0000 | 3.0000 | 4.2 (21) |
|  | *Ho* | 0.1379 | 0.2857 | 0.4828 | 0.3448 | 0.2069 | 0.2916 |
|  | *He* | 0.7326 | 0.3818 | 0.8040 | 0.7108 | 0.1918 | 0.5642 |
|  | *Fis* | **0.8144** | 0.2552 | **0.4038** | **0.5193** | -0.0804 | **0.4886** |
| HaiN |  |  |  |  |  |  |  |
|  | *N* | 42 | 40 | 42 | 42 | 42 | 42 |
|  | *Na* | 6.0000 | 2.0000 | 7.0000 | 4.0000 | 3.0000 | 4.4 (22) |
|  | *Ho* | 0.0952 | 0.0000 | 0.5238 | 0.3810 | 0.3810 | 0.2762 |
|  | *He* | 0.7329 | 0.0974 | 0.7329 | 0.6492 | 0.3821 | 0.5189 |
|  | *Fis* | **0.8728** | **1.0000** | **0.2903** | **0.4192** | 0.0031 | **0.4730** |
| HuN |  |  |  |  |  |  |  |
|  | *N* | 84 | 84 | 78 | 84 | 84 | 83 |
|  | *Na* | 7.0000 | 7.0000 | 9.0000 | 4.0000 | 2.0000 | 5.8 (29) |
|  | *Ho* | 0.0000 | 0.3333 | 0.4359 | 0.2857 | 0.0238 | 0.2158 |
|  | *He* | 0.5014 | 0.6386 | 0.8202 | 0.6394 | 0.0238 | 0.5247 |
|  | *Fis* | **1.0000** | **0.4810** | **0.4718** | **0.5562** | -0.0000 | **0.5945** |
| HuB |  |  |  |  |  |  |  |
|  | *N* | 90 | 88 | 90 | 90 | 88 | 89 |
|  | *Na* | 6.0000 | 3.0000 | 8.0000 | 5.0000 | 4.0000 | 5.2 (26) |
|  | *Ho* | 0.1556 | 0.4091 | 0.6000 | 0.7333 | 0.3182 | 0.4432 |
|  | *He* | 0.7054 | 0.4734 | 0.8287 | 0.6859 | 0.3085 | 0.6004 |
|  | *Fis* | **0.7814** | **0.1371** | **0.2783** | -0.0700 | -0.0317 | **0.2650** |
| XJ |  |  |  |  |  |  |  |
|  | *N* | 80 | 80 | 78 | 78 | 78 | 79 |
|  | *Na* | 3.0000 | 3.0000 | 7.0000 | 5.0000 | 3.0000 | 4.2 (21) |
|  | *Ho* | 0.0250 | 0.4750 | 0.7949 | 0.7179 | 0.1538 | 0.4333 |
|  | *He* | 0.5630 | 0.4997 | 0.7393 | 0.7116 | 0.1915 | 0.5410 |
|  | *Fis* | **0.9550** | -0.0256 | -0.0632 | -0.0293 | **0.1971** | **0.1896** |
| YN |  |  |  |  |  |  |  |
|  | *N* | 52 | 52 | 52 | 52 | 52 | 52 |
|  | *Na* | 3.0000 | 2.0000 | 7.0000 | 3.0000 | 1.0000 | 3.2 (16) |
|  | *Ho* | 0.0000 | 0.6538 | 0.5769 | 0.6923 | 0.0000 | 0.3846 |
|  | *He* | 0.2745 | 0.4487 | 0.7134 | 0.5520 | 0.0000 | 0.3977 |
|  | *Fis* | 1.0000 | -0.4857 | 0.1755 | -0.2787 | - | **0.0336** |

-, *Fis* could not be calculated because *H*o and *He* were zero. The number in the parenthesis is the total number of the alleles.
